# Differential Adverse Event Profiles Associated with BCG as a Preventive Tuberculosis Vaccine or Therapeutic Bladder Cancer Vaccine Identified by Comparative Ontology-Based VAERS and Literature Meta-Analysis

**DOI:** 10.1371/journal.pone.0164792

**Published:** 2016-10-17

**Authors:** Jiangan Xie, Christopher Codd, Kevin Mo, Yongqun He

**Affiliations:** 1 Key Laboratory of Dependable Service Computing in Cyber Physical Society, Ministry of Education, College of Computer Science, Chongqing University, Chongqing, China; 2 Unit for Laboratory Animal Medicine, Department of Microbiology and Immunology, Center for Computational Medicine and Bioinformatics, University of Michigan Medical School, Ann Arbor, Michigan, United States of America; 3 College of Literature, Science, and the Arts, University of Michigan, Ann Arbor, Michigan, United States of America; University of Delhi - South Campus, INDIA

## Abstract

*M*. *bovis* strain Bacillus Calmette–Guérin (BCG) has been the only licensed live attenuated vaccine against tuberculosis (TB) for nearly one century and has also been approved as a therapeutic vaccine for bladder cancer treatment since 1990. During its long time usage, different adverse events (AEs) have been reported. However, the AEs associated with the BCG preventive TB vaccine and therapeutic cancer vaccine have not been systematically compared. In this study, we systematically collected various BCG AE data mined from the US VAERS database and PubMed literature reports, identified statistically significant BCG-associated AEs, and ontologically classified and compared these AEs related to these two types of BCG vaccine. From 397 VAERS BCG AE case reports, we identified 64 AEs statistically significantly associated with the BCG TB vaccine and 14 AEs with the BCG cancer vaccine. Our meta-analysis of 41 peer-reviewed journal reports identified 48 AEs associated with the BCG TB vaccine and 43 AEs associated with the BCG cancer vaccine. Among all identified AEs from VAERS and literature reports, 25 AEs belong to serious AEs. The Ontology of Adverse Events (OAE)-based ontological hierarchical analysis indicated that the AEs associated with the BCG TB vaccine were enriched in immune system (e.g., lymphadenopathy and lymphadenitis), skin (e.g., skin ulceration and cyanosis), and respiratory system (e.g., cough and pneumonia); in contrast, the AEs associated with the BCG cancer vaccine mainly occurred in the urinary system (e.g., dysuria, pollakiuria, and hematuria). With these distinct AE profiles detected, this study also discovered three AEs (i.e., chills, pneumonia, and C-reactive protein increased) shared by the BCG TB vaccine and bladder cancer vaccine. Furthermore, our deep investigation of 24 BCG-associated death cases from VAERS identified the important effects of age, vaccine co-administration, and immunosuppressive status on the final BCG-associated death outcome.

## Introduction

Bacillus Calmette–Guérin (BCG), a live attenuated strain of *Mycobacterium bovis*, has been the only officially registered human vaccine against tuberculosis (TB) for almost a century [1, 2]. TB is a serious infectious disease induced by Gram-positive bacterium *M*. *tuberculosis* that is estimated to cause more than 2 million human deaths each year. In 2014, there were 9.6 million new cases of TB reported globally, with 5.4 million cases in adult males, 3.2 million in adult females, and 1 million in children. The World Health Organization (WHO) recommends that the BCG vaccine should be given to all infants as soon as possible after birth in countries with a high burden of TB [3].

In addition to be a TB vaccine, BCG can also serve as a bladder cancer immunotherapeutic vaccine [4, 5]. In 1929, Pearl published his autopsy study results demonstrating a lower frequency of cancer in patients with TB [6]. Since then many studies have showed the benefits of BCG usage against bladder cancer [5]. In 1990, the US Food and Drug Administration (FDA) approved the general use of intravesical BCG in patients with superficial bladder cancer. Over 20 years of usage have further proven the efficiency and superior status of the BCG therapy compared to other intravesical agents for bladder cancer.

Despite the usefulness of the BCG vaccine in preventing TB and treating bladder cancer, BCG vaccination has been reported to be associated with various adverse events (AEs) including some serious AEs (SAEs) [7–9]. As shown in the literature, the most commonly reported AEs for the BCG vaccine include injection site abscess, lymphadenitis, and severe local reactions [7]. The most severe AE associated with the BCG vaccine is death [8]. Besides literature records, post-licensure safety surveillance programs have been a major source of reporting and collecting BCG vaccine AEs. In the US, the Vaccine Adverse Event Reporting System (VAERS) is a spontaneous vaccine AE case reporting system used to report and monitor AE cases associated with all licensed vaccines [10]. VAERS has received AE reports from health care providers, vaccine recipients or their parents, vaccine manufacturers, and other interested parties. Since 1990’s, VAERS has collected millions of vaccine AE case reports. Although VAERS data does not support the identification of AE causality, statistical VARES data analysis has provided ways to develop hypotheses on AE causality and has greatly supported AE studies and vaccine safety [11].

No study has been reported to compare the AEs associated with the BCG TB vaccine and the BCG therapy for bladder cancer. The BCG TB vaccine is typically given in the infants who are at a healthy condition. In contrast, the BCG cancer therapy is mainly used for adults or geriatric patients who have been diagnosed with bladder cancer. Although we expect that different AE profiles may be resulted from these two types of vaccinations, VAERS categorizes these two vaccination types under the same “BCG” and does not separate them based on the purpose of the vaccination. Such a combined categorization can be confusing. However, since the same live attenuated BCG vaccine strain is used, it would be very interesting to identify the exact AE profiles associated with these two different types of vaccinations. We hypothesized that preventive BCG TB vaccination and therapeutic BCG cancer treatment would result in many distinct AE profiles as well as some shared AEs in administered human populations. Such a hypothesis is addressed in the current study.

To analyze AEs from different resources, it is critical to normalize the results using a standard controlled terminology. The Medical Dictionary of Regulatory Activities (MedDRA) is an international medical terminology dictionary used by regulatory authorities in the pharmaceutical industry during regulatory processes including AE result presentation and evaluation [12]. MedDRA is the standard controlled terminology system used by the VAERS vaccine AE reporting and analysis. However, MedDRA has many issues by itself including its lack of term definitions, poor hierarchical classification, and lack of robust relations among AEs [13]. To address these issues, a community-based biomedical ontology in the domain of AE terminology − Ontology of Adverse Events (OAE) has been developed [14]. A biomedical ontology is a human- and computer-interpretable set of terms and relations that represent entities in a specific biomedical domain and how these entities relate to each other. After an AE term mapping between MedDRA and OAE, OAE can be used to classify VAERS reported AEs represented with MedDRA terms [15, 16]. Empirical evidences have shown that OAE performs better than MedDRA in the area of AE classification [15, 17].

Although many literature papers and the VAERS database have provided various reports of AEs associated with the BCG vaccine, a systematic comparative study on the AEs associated with the BCG as a preventive TB vaccine or therapeutic bladder cancer vaccine has not been reported. The objective of this project is based on OAE classification method to systematically collect and comparatively analyze various AE data associated with these two types of BCG vaccine that extracted from the VAERS database and PubMed literature.

## Materials and Methods

### BCG Adverse Event Data Extraction from VAERS

Using the CDC Wonder data access program (http://wonder.cdc.gov/vaers.html), the VAERS database was searched for all BCG-associated AE case reports. Current VAERS database allows query of AE case report data starting from 1990. In order to include every possible case report for BCG, we queried AE case reports from all locations, and the AE reported time was set from July 1990 through May 2016. Categories of search included symptom, vaccine, age, gender, territory, and VAERS ID.

The BCG vaccine category under VAERS database includes three subcategories: “BCG (MYCOBAX)”, “BCG (NO BRAND NAME)”, and “BCG (TICE)”. Since VAERS does not label each AE case report involved in a BCG TB preventive or bladder cancer therapeutic vaccination, we manually checked every BCG AE case report and identified the vaccine type for each case report based on the administration route and other descriptions in the each AE case report.

### Statistical analysis with PRR, Chi-square test, and base level filtration

To identify statistically significant AEs, three standard methods were applied, including the Proportional Reporting Ratio (PRR) [18], Chi-square test [19], and base level filtration [20]. PRR is a statistical method used to measure if an AE is more likely to result from one specific vaccine (or a vaccine type) than from the whole class of vaccines. Chi-square is used to determine whether there is a significant difference between observed values and expected values in an area of interest. A Chi-square score of 4 is equivalent to a p-value of 0.05. Base level filtration usually used a minimal sample size cutoff for filtering out background noise. Specifically, to be classified as a statistically significant AE, three criteria had to be met: minimal case report number ≥ 3, PRR score ≥ 2, and Chi-square score ≥ 4 [18, 20].

### OAE- or MedDRA-based AE group classification

For MedDRA-based AE classification, the hierarchical structure of AE terms was extracted from the BioPortal MedDRA website (https://bioportal.bioontology.org/ontologies/MEDDRA, accessed at 1st June, 2016) and displayed with the Protégé-OWL editor [21]. For OAE-based AE classification, statistically significant AE terms were first mapped from MedDRA to OAE. After the MedDRA-OAE term mapping, the subset hierarchies that include BCG vaccine type-specific AEs and their parent terms were extracted from OAE using the OntoFox program [22]. The hierarchical structures of these terms were also visualized using the Protégé-OWL editor.

### Examination of BCG-associated death AE in VAERS

To in-depth study BCG-associated death AE in VAERS database, vaccinee’s age, territory, gender, time elapsed, co-administered vaccines, and current illness in each death AE case report were all further investigated to understand whether any of these factors might significantly influence the chances of vaccinee suffering this AE. The values of these variables in each case report indicating death or sudden death was extracted and studied.

### Meta-analysis of BCG-associated AEs from PubMed literature

A meta-analysis of previous written studies on BCG-associated AEs was performed by following the PRISMA (Preferred Reporting Items for Systematic Reviews and Meta-Analyses) guidelines [23, 24]. The PRISMA checklist is provided in S1 File. Briefly, the meta-analysis was done by searching the PubMed database (http://www.ncbi.nlm.nih.gov/pubmed) with the search keywords (‘BCG’ and ‘adverse events’). The abstracts and full text of the articles were retrieved and annotated independently by two reviewers, and any disagreements resolved by discussions and analysis with a third reviewer. Those articles without any related real AE data were excluded from further study. In the eligible papers, BCG-related AEs and BCG vaccine types were manually identified, extracted, and analyzed.

## Results

### Identification and differentiation of 397 VAERS BCG AE case reports associated with 857 unique AEs

From July 1990 through May 2016, our query of the VAERS database identified 397 AE case reports for BCG usage. After manual checking of each of 397 BCG AE case reports, we found that 47 BCG AE reports under the BCG vaccine subcategory “BCG (MYCOBAX)” were associated with the usage of BCG as the TB vaccine, and only 6 reports associated with the cancer vaccine. Meanwhile, we found that 52 reports under the subcategory “BCG (TICE)” were related to the use of BCG as the cancer vaccine, and 24 related to the TB vaccine. The BCG subcategory of “BCG (NO BRAND NAME)” included 217 case reports with the TB vaccine and 51 with the cancer vaccine. In total, 288 AE case reports were associated with BCG as a preventive TB vaccine and 109 AE case reports associated with BCG as a therapeutic cancer vaccine. The detailed VAERS case report IDs and their associated vaccine types are recorded in the S2 File.

All the AE symptoms in each AE case report were collected and matched to the MedDRA AE codes. In total, 857 unique AEs with their corresponding MedDRA terms were identified from all the 397 case reports. It is cautious here that the identification of these AEs does not mean that each of these AEs is caused by BCG. It only means that these AEs occur shortly after the BCG vaccination, and the temporal association between the vaccination and the AE may or may not be causally related to the vaccination in individual patients.

### Statistical analysis of BCG-associated AE terms using VAERS data

Of all 857 BCG AEs, 206 had minimal 3 case reports associated with either the BCG TB vaccine or the BCG bladder cancer vaccine. Out of these 206 AEs, 47 AEs (e.g., pathology test and hemoglobin normal) were ambiguous or not considered to be AEs and then be excluded for further analysis. By adopting the screening criteria include PRR score (≥ 2) and Chi-square score (≥ 4), our statistical analysis eventually identified 64 and 14 AEs significantly associated with the BCG as TB vaccine (Table 1 and S1 Fig) and bladder cancer vaccine (Table 2 and S2 Fig), respectively. Complementary to Tables 1 and 2, S1 and S2 Figs provide intermediate level terms in the ontological hierarchy of different AEs.

**Table 1:** Sixty-four statistically significant AEs associated with BCG as TB vaccine.

Adverse Event	Count	PRR	Chi-square
**behavioral and neurological AE (5)**			
chills	6	7.65	34.63
cold sweat	3	3.51	5.38
depression	3	5.07	9.78
irritability	9	2.30	6.68
psychomotor retardation	5	164.92	750.07
**cardiovascular AE (5)**			
bradycardia	3	5.55	11.18
epistaxis	3	6.23	13.14
hemorrhage	4	9.15	28.96
hypertension	4	3.48	7.10
tachycardia	4	2.75	4.48
**digestive AE (6)**			
gastroenteritis*	4	8.01	24.49
hematochezia*	10	9.40	74.94
intussusception*	7	6.81	34.66
diarrhea	18	2.26	13.03
feces discolored	3	10.38	25.31
mucous stool	3	11.39	28.28
**gustatory system AE (1)**			
hypophagia	5	8.97	35.33
**hematopoietic system AE (4)**			
anemia	8	12.70	85.86
sedimentation rate increased	8	8.22	50.72
splenomegaly	3	28.84	79.46
thrombocytopenia	4	5.92	16.34
**hepatobiliary or pancreatic AE (1)**			
hepatomegaly*	4	30.25	111.41
**immune system AE (8)**			
hepatosplenomegaly*	3	74.54	209.52
immunodeficiency*	3	98.95	276.61
meningitis*	3	4.63	8.53
abscess	10	26.68	244.11
allergy	4	4.51	10.94
granuloma	9	136.65	1131.39
lymphadenitis	12	67.52	760.10
lymphadenopathy	32	8.21	204.38
**infection AE (3)**			
disseminated BCG infection*	14	13391.58	23432.45
tuberculosis*	10	910.99	6158.04
respiratory tract infection	3	21.34	57.53
**investigation result abnormal AE (6)**			
aspartate aminotransferase level increased	3	3.46	5.26
blood bilirubin level increased	3	12.50	31.57
blood creatinine level decreased	3	10.72	26.34
C-reactive protein (CRP) increased	6	4.90	18.66
hemoglobin level decreased	3	4.33	7.68
hypocalcemia	3	95.65	267.64
**metabolism, endocrine or exocrine system AE (1)**			
necrosis	3	38.779	108.25
**musculoskeletal or connective tissue AE (3)**			
osteomyelitis*	8	170.05	1234.93
hypotonia	9	2.87	11.09
joint swelling	4	3.96	8.84
**respiratory system AE (8)**			
apnea*	3	6.90	15.10
pneumonia*	11	6.53	51.61
respiratory failure*	6	16.73	87.73
cough	30	4.52	83.84
lung disorder	5	11.68	48.60
nasal congestion	4	3.23	6.17
respiratory disorder	8	14.86	102.81
respiratory rate increased	17	59.13	943.37
**serious AE (3)**			
death*	17	13.48	195.97
disability*	5	40.53	188.86
sudden death*	6	93.32	522.63
**skin AE (8)**			
cyanosis	7	3.04	9.61
flushing	4	2.88	4.92
skin discoloration	4	2.66	4.17
skin exfoliation	3	8.25	19.04
skin lesion	5	4.10	11.72
skin mass	4	4.46	10.73
skin ulceration	7	4.68	20.32
thrombocytopenic purpura	3	9.33	22.23
**syndrome AE (1)**			
cachexia*	3	168.80	459.88
**tumor AE (1)**			
axillary mass	4	55.86	209.44

Note:

* = serious adverse event (SAE). Specific AEs are labeled with MedDRA terms. The top-level categories follow the OAE hierarchy.

**Table 2:** Fourteen statistically significant AEs associated with BCG as bladder cancer vaccine.

Adverse Event	Count	PRR	Chi-square
**behavioral and neurological AE (3)**			
back pain	6	5.27	20.93
chills	17	4.80	52.86
skin burning sensation	5	38.82	183.06
**infection AE (1)**			
bacterial infection	4	15.74	55.15
**investigation result abnormal AE (1)**			
urine analysis result abnormal	3	20.30	54.90
**musculoskeletal or connective tissue AE (1)**			
arthralgia	9	2.83	10.94
**syndrome AE (1)**			
flu-like syndrome	10	11.01	91.58
**systematic AE (1)**			
condition aggravated	4	3.79	8.30
**urinary system (6)**			
hematuria*	13	167.21	2080.51
urinary incontinence*	3	16.72	44.26
dysuria	20	227.18	4313.62
pollakiuria	14	176.92	2367.87
urgent urination	5	227.69	1080.07
urinary tract infection	5	24.63	113.03

Note:

* = serious adverse event (SAE). Specific AEs are labeled with MedDRA terms. The top-level categories follow the OAE hierarchy.

The 64 AEs associated with the TB vaccine were categorized into 16 OAE AE classes (Table 1). Based on the case report numbers, the most reported AEs were lymphadenopathy (32 reports), following by cough (30 reports) and diarrhea (18 reports). The most frequently observed AE groups were ‘immune system AE’, ‘respiratory system AE’, and ‘skin AE’, each with 8 specific AE classes. The 14 statistically significant AEs associated with the bladder cancer vaccine were enriched in the urinary system (Table 2 and S2 Fig). There were 6 urinary system AEs, including hematuria, urinary incontinence, dysuria, pollakiuria, urgent urination, and urinary tract infection. In addition, the urine analysis result abnormal is also related to the urinary system AE.

### AE profiles associated with BCG vaccine based on literature meta-analysis

**Fig 1:**
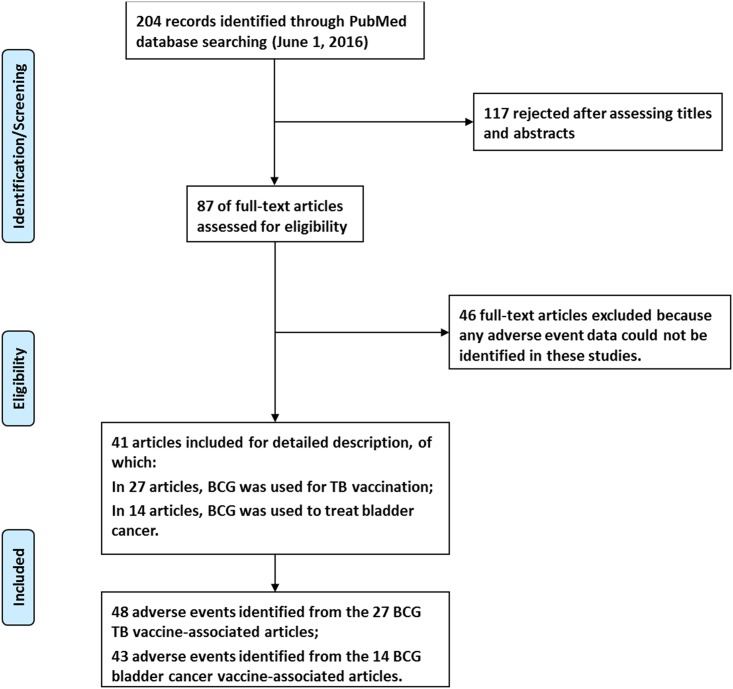
PRISMA flowchart of the selection of relevant papers.

The inclusion criteria for selection of eligible articles from PubMed and the final results are provided in the PRISMA flowchart (Fig 1). The selection criteria are two-fold. First, the articles were all searchable in PubMed by using the keywords “BCG” and “adverse events”. With this criterion, our literature search identified 204 potentially eligible articles on June 1, 2016. The second article inclusion criterion is the identification from the article of specific AEs detected from randomized controlled trials or spontaneous case report studies. With this criterion, the number of related articles was reduced to 87. From these 87 articles, our PubMed literature meta-analysis eventually identified a total of 41 peer-reviewed journal articles that contained specific BCG-associated AEs. The details of these articles and the AEs identified from each articles are provided in S3 File. Note that we have examined all the 41 eligible peer-reviewed articles in PubMed as well as the VAERS BCG-associated AE case reports, and we have not found any information in terms of latent tuberculosis screening in these case reports and articles.

In 27/41 (65.9%) of these articles, TB vaccination was the reason for BCG usage; in the remaining 14/41 (34.1%), BCG was used to treat bladder cancer. From these papers, we identified 48 AEs associated with BCG as a TB vaccine (Table 3 and S3 Fig) and 43 AEs were associated with BCG as a cancer vaccine (Table 3 and S4 Fig). The comparison of these results clearly show significant differences in the AE profiles associated with the two BCG vaccine types. For example, compared with the cancer vaccine, the TB vaccine was significantly more associated with skin AEs (11 vs 1) and lymphatic immune system AEs (6 vs 0) (Table 3 and S3 Fig). In particular, there had no report on any urinary system AE associated with the TB vaccine (Table 3). However, 14 urinary system AEs associated with the cancer vaccine were reported (Table 3).

**Table 3:** BCG-associated AEs from literature meta-analysis.

AEs associated with BCG used as TB vaccine	AEs associated with BCG used as bladder cancer vaccine
**behavioral and neurological AE (8)**	**behavioral and neurological AE (7)**
dizziness	chills
fatigue	headache
gastrointestinal pain	kidney pain
headache	lower abdominal pain
malaise	malaise
musculoskeletal pain	miction pain
myalgia	urethral pain
seizure	**cardiovascular AE (1)**
**cardiovascular AE (2)**	hypertension
hematoma	**digestive system AE (1)**
hemorrhage	diarrhea
**hematopoietic system AE (1)**	**gustatory system (1)**
sedimentation rate increased	anorexia
**homeostasis AE (2)**	**hematopoietic system AE (1)**
edema	pancytopenia
fever	**homeostasis AE (1)**
**immune system AE (14)**	fever
hepatosplenomegaly*	**immune system AE (9)**
meningitis*	hepatitis*
pneumonia*	pneumonia*
vasculitis*	allergy
abscess	bacterial cystitis
axillary lymphadenitis	chemical cystitis
axillary lymphadenopathy	cystitis
hepatic abscess	granuloma
hepatosplenic granuloma	granulomatous cystitis
lymphadenitis	granulomatous prostatitis
lymphadenopathy	**infection AE (3)**
osteitis	sepsis*
staphylococcal abscess	bacterial infection
suppurative lymphadenitis	lung infection
**infection AE (1)**	**investigation result abnormal AE (1)**
disseminated BCG infection*	CRP increased
**investigation result abnormal AE (1)**	**musculoskeletal or connective tissue AE (2)**
CRP increased	arthralgia
**local AE (2)**	asthenia
injection site discharge	**skin AE (1)**
injection site ulcer	rash
**musculoskeletal or connective tissue AE (2)**	**syndrome AE (1)**
osteomyelitis*	flu-like syndrome
asthenia	**urinary system AE (14)**
**nervous system AE (1)**	hematuria*
paresthesia	macroscopic hematuria*
**serious AE (1)**	urinary incontinence*
death*	bladder atrophy
**skin AE (11)**	bladder spasm
acute cutaneous erythema	contracted bladder
eczema	dysuria
induration	irritative bladder symptom
keloid	lower urinary tract symptom
papule	nocturia
pruritus	pollakiuria
purpura	pyuria
pustule	urinary frequency
scab	urinary tract infection
scar	
urticaria	
**syndrome AE (2)**	
immune reconstitution inflammatory syndrome*	
Kawasaki disease*	

Note:

* = serious adverse event (SAE). Specific AEs are labeled with MedDRA terms. The top-level categories follow the OAE hierarchy.

Table 4 includes a list of the 12 most commonly reported AEs based on the total numbers of articles reporting individual AEs. Out of these 41 annotated journal articles, the most frequently reported AEs were fever and hematuria, mentioned in 16 and 11 articles, respectively (Table 4). Three AEs (i.e., fever, malaise, and headache) were associated with both BCG vaccine types. Note that among the 6 TB vaccine-specific AEs, injection site ulcer and erythema were reported in VAERS, but they were not statistically significant (so unavailable in Table 1).

**Table 4:** Twelve most commonly reported BCG AEs from the literature.

Adverse event	Count	TB vaccine	bladder cancer vaccine
***AEs shared by TB vaccinees and bladder cancer patients (all in*** Table 1***)*:**			
fever	16 (39%)	6 (37.5%)	10 (62.5%)
malaise	8 (20%)	1 (12.5%)	7 (87.5%)
headache	4 (10%)	3 (75%)	1 (25%)
***AEs only found in TB vaccinees (all in*** Table 1 ***except injection site ulcer and erythema)*:**			
lymphadenitis	10 (24%)	10 (100%)	0 (0%)
injection site ulcer	9 (22%)	9 (100%)	0 (0%)
abscess	8 (20%)	8 (100%)	0 (0%)
disseminated BCG infection	5 (12%)	5 (100%)	0 (0%)
lymphadenopathy	5 (12%)	5 (100%)	0 (0%)
erythema	4 (10%)	4 (100%)	0 (0%)
***AEs only found in bladder cancer patients (all in*** Table 2***)*:**			
hematuria	11 (27%)	0 (0%)	11 (100%)
cystitis	6 (15%)	0 (0%)	6 (100%)
sepsis	5 (12%)	0 (0%)	5 (100%)

**Fig 2:**
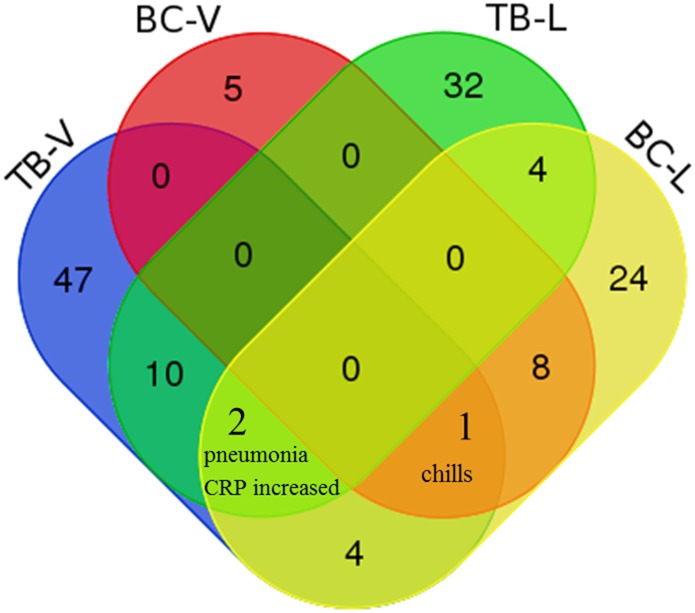
Venn diagram summary of the BCG-associated AEs from VAERS statistical analysis and literature meta-analysis. TB-V: AEs associated with BCG TB vaccine from VAERS statistical analysis. TB-L: AEs associated with BCG TB vaccine from literature meta-analysis. BC-V: AEs associated with BCG bladder cancer vaccine from VAERS statistical analysis. BC-L: AEs associated with BCG bladder cancer vaccine from literature meta-analysis. Three AEs (i.e., chills, pneumonia, and CRP level increased) are shared by the BCG TB vaccine and bladder cancer vaccine.

Fig 2 is a summary Venn diagram that compares four groups of the AEs (Tables 1–3) associated with BCG TB vaccine or bladder cancer vaccine using VAERS and literature resources. It is surprising that none of the AEs was conserved in all the four AE groups. Only three AEs (i.e., chills, pneumonia, and C-reactive protein increased) were shared by the BCG TB vaccine and bladder cancer vaccine (Fig 2). C-reactive protein (CRP) is produced by the liver in response to inflammation. Further investigation found that those AEs shown in the literature but not from VAERS statistical analysis indeed existed in the VAERS case report database. They were not shown in the Tables 1 and 2 because they did not pass the statistical filtering cutoffs. In summary, our comparative study indicates that the BCG TB vaccine and cancer vaccine were associated with quite different AE profiles, the two profiles could be identified from both VAERS statistic data analysis and literature reports although specific lists of AEs might differ from the analyses of these two resources.

### Differential BCG TB and bladder cancer vaccine-specific SAE profiles

Among all the AEs specific to the BCG TB vaccine, 20 unique AEs including 17 AEs from VAERS (Table 1) and 9 AEs from literature reports (Table 3) were further classified as SAEs [9]. For BCG as a bladder cancer vaccine, our meta-analysis identified 6 unique SAEs including 2 SAEs (Table 2) from VAERS and 6 SAEs from literature reports (Table 3). Specifically, pneumonia was the only AE shared by both BCG vaccine types. Overall, a total of 25 SAEs associated with BCG usage.

**Fig 3:**
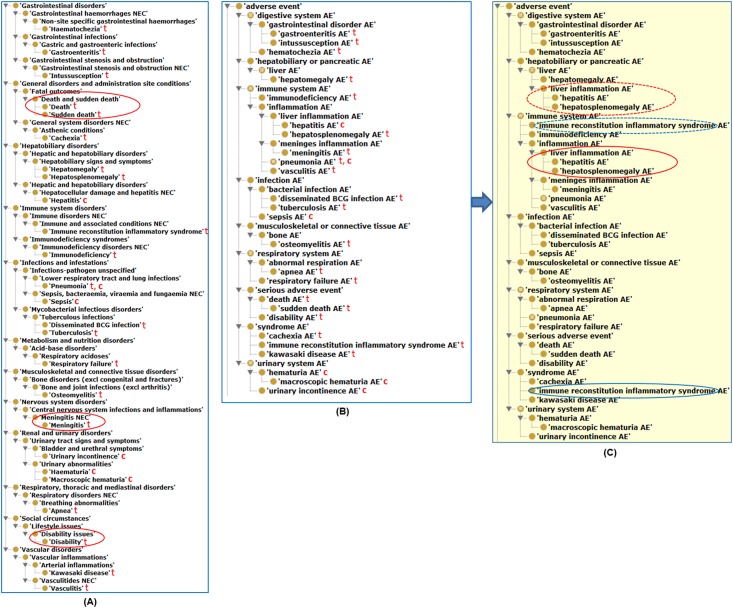
Hierarchical classification of 25 BCG-associated SAEs and their related top level classes using MedDRA and OAE. (A) MedDRA-based classification. All oval circle highlighted terms are discussed in the main text. (B) Asserted OAE hierarchy of the 25 SAEs and related top level classes. (C) Inferred OAE hierarchy after reasoning with the ELK reasoner (version 0.4.10, downloaded from website: https://www.cs.ox.ac.uk/isg/tools/ELK/). The SAE labeled with “t” and “c” represents the SAE associated with BCG as a tuberculosis and bladder cancer vaccine, respectively. After reasoning, two classes of terms were inferred to under different parent terms as highlighted in dotted oval circles.

For better understanding of the SAEs, all the SAEs were classified and compared as subsets of the complete MedDRA and OAE hierarchies (Fig 3). Obviously, the MedDRA subset hierarchy (Fig 3A) appears to be much larger and complicated than the OAE subset hierarchy (Fig 3B). Actually, MedDRA has many drawbacks in terms of AE classification. First, MedDRA includes many terms ended with “NEC” (i.e., “not elsewhere classified”), such as ‘Meningitis NEC’, in its hierarchical structures (Fig 3A). Such an “NEC” term is defined arbitrarily and ambiguously, and its usage often leads to confusing and unclear classification results. Second, MedDRA often misses obvious parent-child term logic. For example, in MedDRA, ‘Sudden death’ is listed as a sibling class of ‘Death’, and both are listed as subclasses of ‘Death and sudden death’ (Fig 3A). This is confusing and logically incorrect since a sudden death should be a special subtype of death. Additionally, MedDRA also contains many redundant terms, such as the terms ‘Disability issues’ and ‘Disability’ (Fig 3A). In comparison, all these drawbacks have been avoided in the OAE ontology (Fig 3B).

As a biomedical ontology, OAE supports asserted hierarchy and inferred hierarchy. An asserted hierarchy is the hierarchy asserted by OAE developers. An inferred hierarchy is one after the execution with an ontology reasoner [25]. Many AE terms are often classified under two or more parent terms. For example, ‘immune reconstitution inflammatory syndrome AE’ is a subclass of ‘immune system AE’ or ‘syndrome AE’ (Fig 3B and 3C). The approach of asserting more than one parent term in ontology is called multiple inheritance [26], which often makes an ontology difficult to maintain and update. To avoid the multiple inheritance shortcomings, OAE asserts only one parent term and allows the other term(s) to be inferred as parent(s) through semantic reasoning. In the above example, ‘immune reconstitution inflammatory syndrome AE’ is asserted as a subclass of ‘syndrome AE’ (Fig 3B). After reasoning (based on the internal logical axiom definition of this AE occurring in the immune system), this term is inferred to be a subclass of ‘immune system AE’ as well (Fig 3C). Such a feature does not exist in MedDRA.

The OAE hierarchical classification (Fig 3B and 3C) clearly shows the patterns of the 25 SAEs associated with the BCG TB vaccine and the cancer vaccine. Among the 6 BCG bladder cancer vaccine-associated SAEs, three (i.e., hematuria AE, macroscopic hematuria AE, and urinary incontinence AE) all belong to the urinary system AE. The hepatitis and pneumonia AEs are both inflammation AEs. Sepsis AE is a special infection AE, likely due to the BCG infection. Among the 20 SAEs associated with the BCG TB vaccine, 6 belong to immune system AEs (Fig 3C), and 4 of these 6 immune system AEs show different types of inflammation. In addition to the immune system, 20 SAEs were found to occur in the digestive and respiratory systems (Fig 3B and 3C). Interestingly, none of the 20 SAEs is related to the urinary system.

### Special profiles associated with 24 BCG-associaed death AE

As of June 1, 2016, VAERS reported 1,272 total death events associated with 101 vaccines, with the average of approximately 12 deaths each vaccine. For the BCG vaccine, a total of 24 death cases (including 6 sudden death cases) were reported (Table 5). This number of BCG-associated deaths doubles the average of 12 deaths per vaccine reported in VAERS. Since death is the most severe AE, we investigated each of BCG-associated death cases with a focus on identifying contributions of different variables to the death outcome (Table 5).

**Table 5:** BCG-associated death AE case reports from VAERS.

VAERS ID	Age (years)	Territory	Gender	Time elapsed	Co-administered vaccines	Current illness
**18 death cases**						
318242	<0.5	Foreign	Female	4–5 hours	DTaP, Hib, PCV7, IPV	Rhinorrhea
353252	0.5–1	Foreign	Male	7 months	None	N/A
368158	<0.5	Foreign	Female	< 2 days	HepB	N/A
383583	3–5	Foreign	Male	N/A	None	Chronic granulomatous disease
383596	<0.5	Foreign	Male	N/A	None	SCID, FHID
383598	<0.5	Foreign	Male	N/A	None	Immunodeficiency disorder
383599	0.5–1	Foreign	Female	N/A	None	SCID
383601	<0.5	Foreign	Female	N/A	None	SCID, FHID
383602	<0.5	Foreign	Female	N/A	None	SCID, FHID
383604	<0.5	Foreign	Male	N/A	None	SCID
383634	<0.5	Foreign	Female	N/A	None	Immunodeficiency disorders, FHID
431084	<0.5	Foreign	Female	1.5 hours	HEP B	N/A
488670	<0.5	Foreign	Female	< 1 day	DTaP+Hib+IPV, HepB, PCV7, IPV, PRV	HIV+, Premature birth, Respiratory distress, Vomiting
491427*	65+	Foreign	Male	< 1 day	None	Bladder cancer
522465	0.5–1	Foreign	Male	< 1 day	DTaP + IPV, HepB, PRV	Asteatosis, Eczema
530241	40–49	Michigan	Female	N/A	DTP	N/A
533126	<0.5	Foreign	Female	>107 days	HepB	N/A
549045	0.5–1	Unknown	Male	N/A	OPV	SCID
**6 sudden death cases**						
414170	<0.5	Foreign	Male	2 days	DTaP + IPV +Hib, PRV	N/A
414704	<0.5	Foreign	Male	2 d	DTaP + IPV +Hib, PRV	N/A
418242	<0.5	Foreign	Male	2 d	Hib	N/A
431084	<0.5	Foreign	Female	1.5 h	HepB	N/A
433276	<0.5	Foreign	Male	N/A	HepB	N/A
433278	<0.5	Foreign	Male	N/A	HepB	N/A

Note:

*The only one case where BCG vaccine was used for bladder cancer treatment. In all other 23 cases, BCG was used as a TB vaccine. SCID: severe combined immunodeficiency; FHID: family history of immunodeficiency.

As shown in Table 5, out of 12 cases with reported time relapsed, 10 cases reported patient death within 2 days after BCG vaccination. The fact that 21 of 24 (87.5%) cases occurred in patients younger than one year old indicates patient age was an important factor for death outcome. Gender was unlikely an important factor since these 24 cases were almost evenly split with 11 females and 13 males. Most of patients were suffering some current illness, especially severe combined immunodeficiency (SCID) and family history of immunodeficiency (FHID), when they were vaccinated with BCG vaccine. Therefore, personal health issue likely influenced the death outcome for BCG vaccination. Twelve patients were co-immunized with other vaccines, including HepB (8 patients), DTAP (3 patients), IPV (5 patients), PCV7 (2 patients), PRV (4 patients), Hib (5 patients), and OPV (one patient). Furthermore, out of 24 death cases, 22 cases were occurred at territory with “foreign” (i.e., countries outside of the USA); the rest of two cases, one occurred in an “Unknown” territory, and the other occurred in Michigan, USA.

## Discussion

The data resources and methods used in our study are relatively novel. To our best knowledge, our paper is the first report focusing on the systematic analysis of AE profiles associated with two different types of BCG vaccine. Our study is also the first to utilize the VAERS database for BCG AE data analysis. One difficulty in our data collection is that VAERS does not differentiate the type (for TB prevention or cancer treatment) of usage for the BCG vaccine. Therefore, we had to manually check each case report to identify the vaccine type. It would be helpful for future studies if clear differentiation of the BCG vaccine type is made in VAERS. Meanwhile, we also collected and analyzed related papers from the PubMed literature database. Compared to providing lists of BCG AEs from other BCG AE papers, our ontology-based AE analysis allowed classification of AEs, leading to more specific insights.

Our comparative results on the differential AE profiles associated with two types of BCG vaccine not only keep consistent with the results from existing reports, but also contain more specific details. The US FDA has reported many BCG AEs based on randomized and well-controlled critical trials [27, 28]. Specifically, for the BCG vaccination against TB, the FDA package insert document reports many AEs including moderate axillary or cervical lymphadenopathy and induration and subsequent pustule formation at the injection site, and disseminated BCG infection as the most serious complication of BCG TB vaccination [27]. For the intravesical BCG use for cancer treatment, the most common complaints for BCG use were malaise, fatigue and lethargy, fever, and abdominal pain. Some serious but uncommon adverse reactions, such as disseminated sepsis and epididymitis have also been reported [28]. Compared the FDA package insert information, our study identified more AEs coming from spontaneous case reports and literature survey. Many other BCG AE reports are also available. For example, Clothier et al. recently surveyed the BCG AEs from a VAERS-like vaccine AE case report database, and their study identified abscess and lymphadenopathy as two predominant BCG AEs [29]. These two AEs were also found in our study. Retrospective BCG AE studies were also conducted in many other countries, for example, Brazil [30], French [31], Ireland [32], Singapore [33], and China (paper written in Chinese) [34]. The BCG AEs reported in these literature articles were collected in our literature meta-analysis study and analyzed again. Our comparative and ontology-based classification also provides more specific details as shown in the Results section.

There are likely many reasons behind the differential AE profiles associated with the use of BCG as a tuberculosis preventive vaccine or bladder cancer therapeutic vaccine. First, the route of administration appears to be an important factor. When BCG is used for TB vaccination, percutaneous or intradermal route is applied [35, 36]. The percutaneous method is recommended for the vaccination of the US FDA-licensed TICE-BCG [27]. One main reason of the preferred usage of the percutaneous method is that the intradermal method is associated with elevated incidences of AEs such as keloid reactions [35]. In contrast, for bladder cancer treatment, BCG is administered intravesically [28]. With this intravesical administration method, BCG is put into a catheter and injected directly into the bladder. Instilled into the bladder, BCG is able to sensitize T cells, activate macrophages, and induce cytokines, and these cellular responses cooperate to kill cancer cells, reduce tumor recurrences, delay cancer progress, and improve survival [5, 37]. Under this situation, it is reasonable to expect the bladder and its nearby systems to experience most of the complications. Second, patient health conditions likely have an important effect on the AE outcomes. The vaccinees to be vaccinated with the TB vaccine are expected to be healthy before the vaccination. However, the patients to be treated with the bladder cancer vaccine have already had a sick bladder. This weakened bladder is likely more susceptible to more AEs compared to that of healthy people. Thirdly, the age is likely another factor since the BCG TB vaccine is frequently used in children and the BCG bladder cancer vaccine is usually used in adults. How each or a combination of these factors contributes to the AE outcomes deserves further investigation.

Our analysis of 24 reported cases of death from VAERS suggests that three factors, including age, health condition, and vaccine co-administration, contribute to the occurrence of BCG-associated death. Our comparative analysis shows that children, patients co-administered with BCG and other vaccine(s), and immunocompromised patients were more susceptible to the death AE after BCG vaccination (Table 5). Of these death cases, many patients suffered from one or more immune system disorders, especially the Severe Combined Immunodeficiency (SCID) (Table 5). A weakened immune system might greatly increase a person’s chance of experiencing a severe AE such as death. SCID is a primary immune deficiency characterized by a severe defect in both T- and B-lymphocyte systems. The annual incidence of SCID is estimated to be one case per 40,000–100,000 live births [38]. SCID patients have been found to be very sensitive to pathogen infections and vaccinations (e.g., rotavirus vaccines) [38]. Our study indicated that SCID patients were very sensitive to BCG, suggesting the importance of listing SCID as a contraindication for the BCG administration. In fact, the BCG package insert from FDA indicates that BCG vaccine should not be used in infants, children, or adults with severe immune deficiency syndromes [27]. All the death cases associated with the immunocompromised health condition occurred in the territory of “Foreign”, suggesting that the BCG immunization in the “Foreign” territory more likely did not follow the guideline from the package insert document.

Overall, this study allows us to address the hypothesis of differential and shared AEs resulted from the preventive BCG TB vaccination and therapeutic BCG cancer treatment. The above discussion focuses on the differential AEs out of these two types of vaccinations. Meanwhile, our study also shows that these two procedures resulted in three shared AEs, i.e., chills (a feeling of coldness occurring during a high fever), pneumonia, and elevated CRP level in blood (indicating the occurrence of inflammation). These three AEs are not directly related to the local percutaneous or intradermal route for TB vaccination or intravesical site for bladder cancer treatment. Instead, such AEs are out of the systemic effects of the BCG vaccination *in vivo* to vaccinees with different health conditions. These results demonstrate that any of these vaccination routes would result in systemic proinflammatory responses in the blood and lung of human vaccinees. How BCG causes such inflammatory effects from different routes deserves further investigations.

Vaccine AE data standardization and classification have been a major research topic in vaccine safety research [39–41]. Our ontology-based BCG AE study demonstrates the ontological and semantic features of OAE in AE classification. A previous study by Sarntivijai et al. first applied the MedDRA-OAE mapping and the following OAE AE classification to study the AEs associated with live attenuated and killed influenza vaccines [15]. By comparing OAE, MedDRA, and SNOMED-CT [42], the study by Sarntivijai et al. showed many advantages of OAE in AE term classification [15]. By analyzing FAERS-reported drug AEs associated with tyrosine kinase inhibitors and monoclonal antibodies, a recent FDA-led research demonstrated that OAE can serve as a semantic framework to link MedDRA-coded clinical AE phenotypes to biological mechanisms [16]. Our current study provides new empirical evidence on the feasibility and advantages of using OAE in AE classification and clustering analysis after the MedDRA-OAE term mappings. Such a method can also be used to study AEs associated with other licensed vaccines. Currently OAE has a lower coverage of AE terms than MedDRA. To ensure all possible MedDRA-OAE mappings and better support AE classification, an increased coverage of OAE is needed.

The information learned from this study is useful for future clinical or basic research. First of all, although the same live attenuated BCG vaccine is used in both the TB vaccination and bladder cancer treatment, our research demonstrates that these two types of vaccination result in many differential AEs and three shared AEs. Second, for better categorization of the BCG-associated AEs from clinical AE reports, our study suggests the necessity of separating these two vaccination types in VAERS in order to avoid possible misinterpretation of BCG-associated AEs. Third, this study also provides a new standard operating procedure in systematically analyzing AEs using VAERS and literature reports. Furthermore, our OAE and MedDRA comparison provides a strong empirical evidence of the benefits of using OAE for more accurate AE classification and new information discovery.

## Conclusion

In this study, we systematically analyzed and compared differential AE profiles associated with the BCG preventive TB vaccine and therapeutic bladder cancer vaccine using two data resources: the VAERS database and the meta-analyzed PubMed literature. The statistical analysis combined with ontology-based AE classification clearly showed that the BCG for TB prevention and cancer treatment were associated with two distinct AE profiles with minimal overlaps. Specifically, when BCG was used as a preventive TB vaccine, those AEs were enriched in immune system, skin, and respiratory system. When BCG was used as a therapeutic bladder cancer vaccine, the major group of associated AEs occurred in the urinary system. Meanwhile, three AEs (i.e., chills, pneumonia, and CRP level increased) were found to be commonly associated with the two vaccine types. In addition, the detailed analysis of BCG-related death cases found that the death outcome was primarily related to patient age, patient immunosuppressive status, and co-administered vaccines. At last, our comparative study for BCG-associated 25 SAEs empirically confirmed the advantages of OAE over MedDRA in AE classification. Overall, our research results facilitate BCG vaccine safety surveillance and benefit rational design of more secure and effective vaccines, and the research methods used in this study can also be applied to study adverse events associated with other licensed vaccines.

## Supporting Information

S1 Fig OAE-based classification of 64 statistically significant AEs associated with BCG as a TB vaccine. (TIF)

S2 Fig OAE-based classification of 14 statistically significant AEs associated with BCG as a bladder cancer treatment vaccine. (TIF)

S3 Fig Classification of 48 AEs associated with the BCG TB vaccine using OAE. (TIF)

S4 Fig Classification of 43 AEs associated with the BCG cancer vaccine using OAE. (TIF)

S1 File PRISMA checklist. (DOC)

S2 File The detailed information of BCG-associated AE case reports in VAERS. (XLSX)

S3 File The details of 41 peer-reviewed journal articles and BCG-associated AEs identified from these articles. (DOCX)
