# Supplementary figures and images for: Differential Adverse Event Profiles Associated with BCG as a Preventive Tuberculosis Vaccine or Therapeutic Bladder Cancer Vaccine Identified by Comparative Ontology-Based VAERS and Literature Meta-Analysis
Source: PLoS One. 2016 Oct 17;11(10):e0164792. doi: 10.1371/journal.pone.0164792 (PMC5066964; doi:10.1371/journal.pone.0164792)

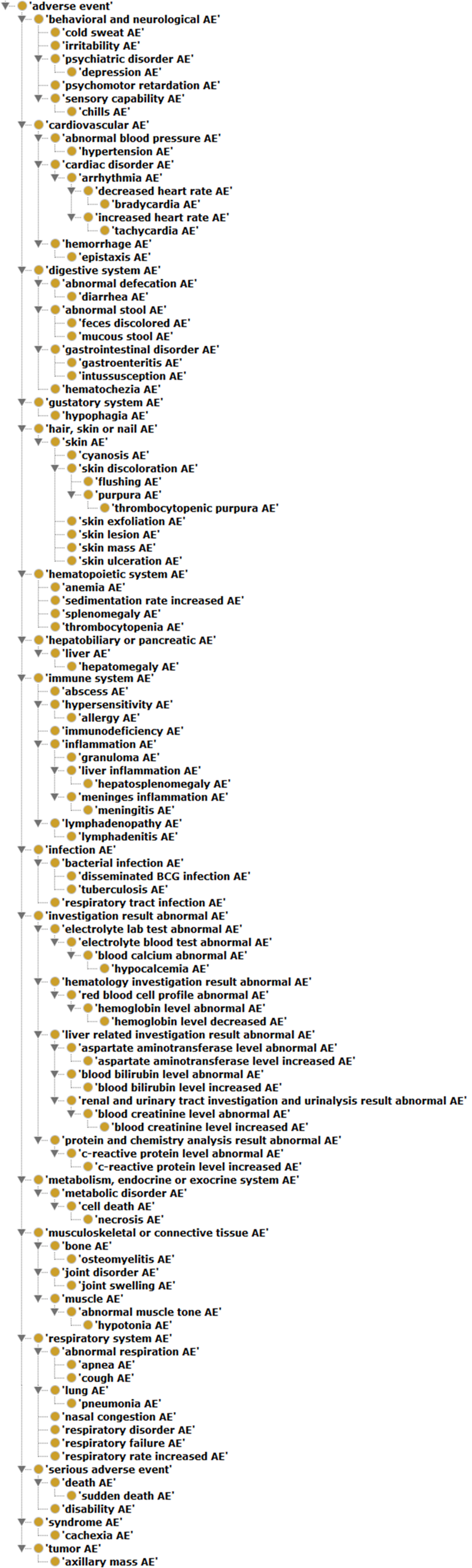

Supplement: S1 Fig — (TIF) [file pone.0164792.s001.tif]

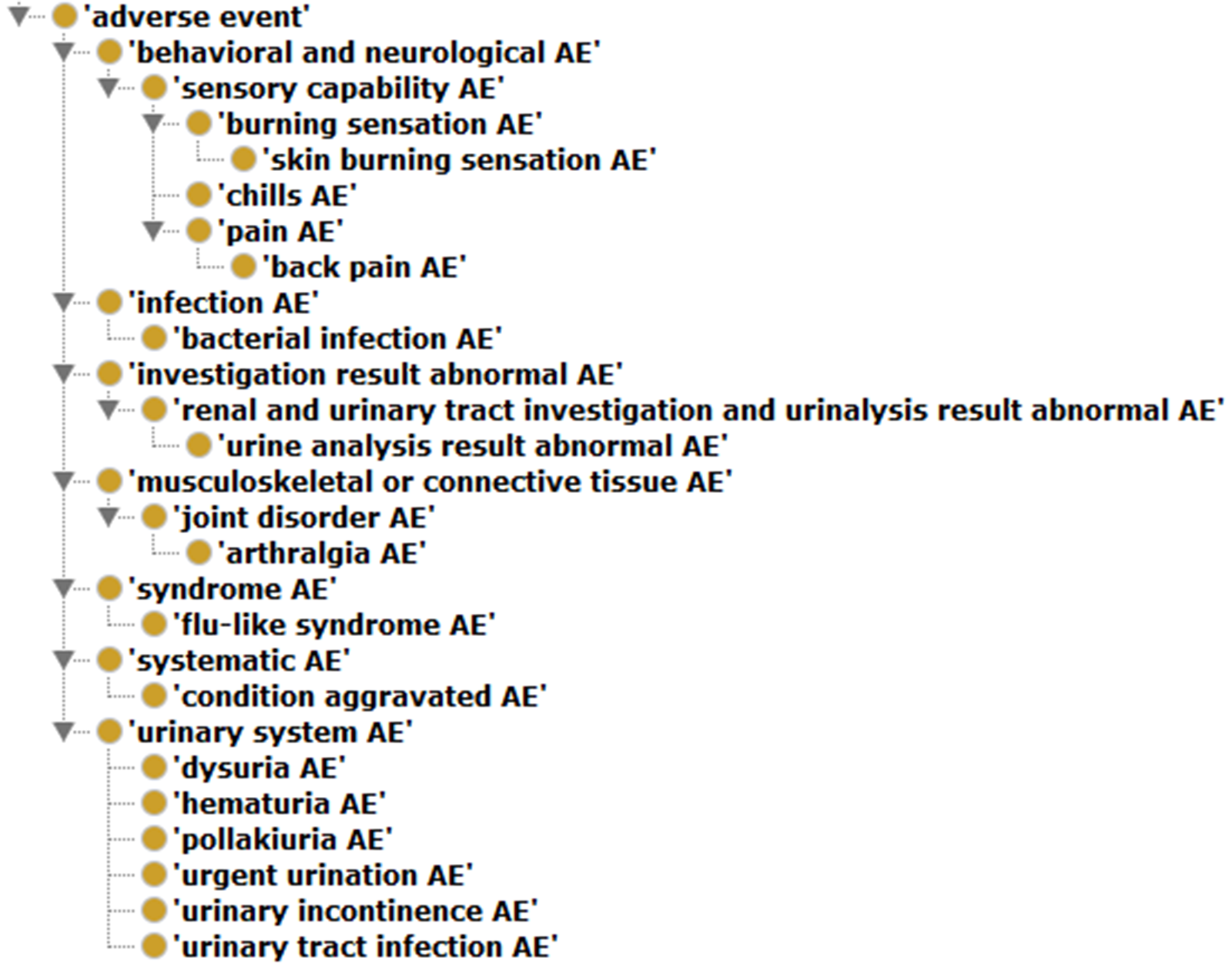

Supplement: S2 Fig — (TIF) [file pone.0164792.s002.tif]

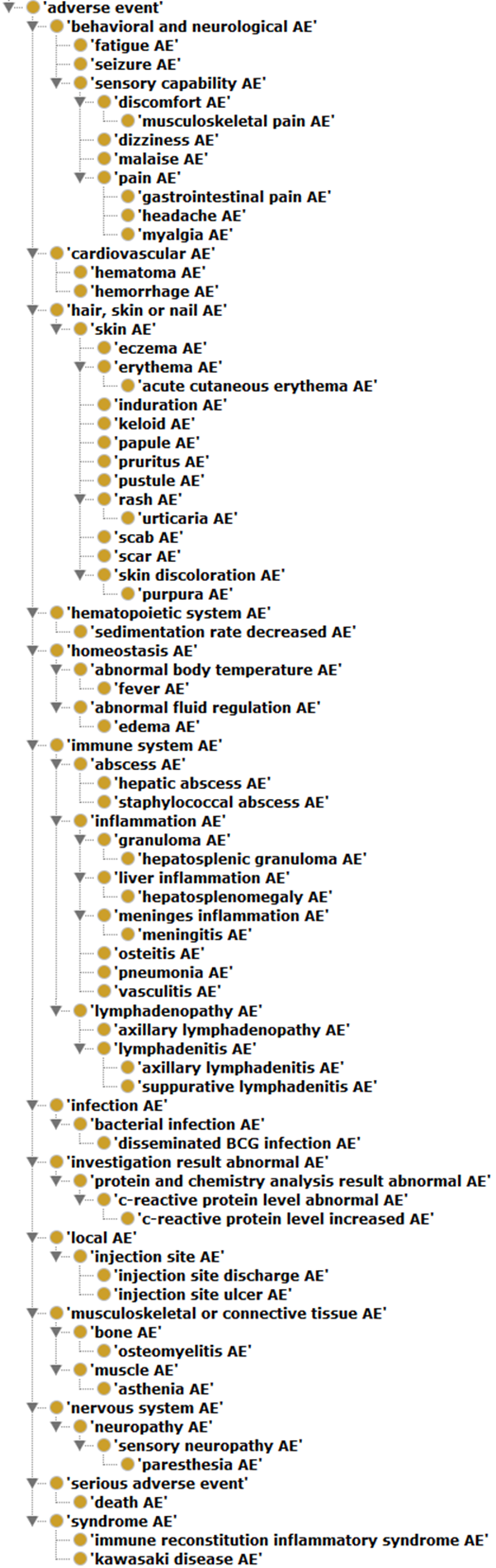

Supplement: S3 Fig — (TIF) [file pone.0164792.s003.tif]

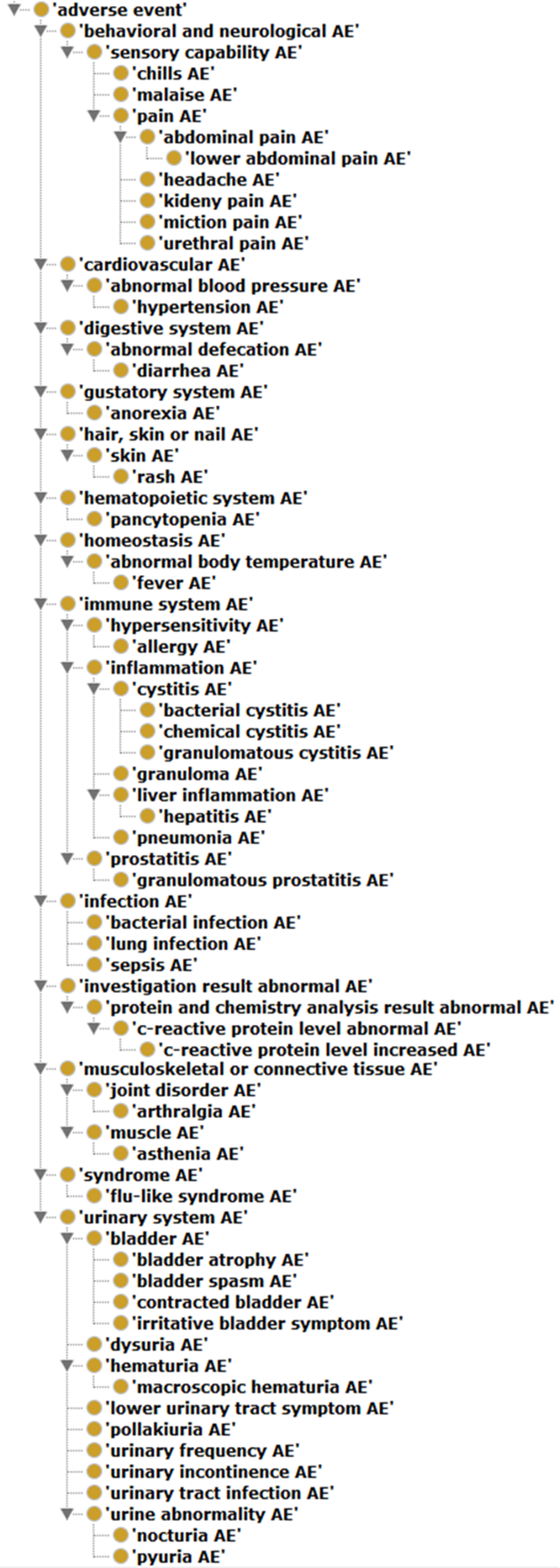

Supplement: S4 Fig — (TIF) [file pone.0164792.s004.tif]
