# Supplementary material for: Differential Adverse Event Profiles Associated with BCG as a Preventive Tuberculosis Vaccine or Therapeutic Bladder Cancer Vaccine Identified by Comparative Ontology-Based VAERS and Literature Meta-Analysis
Source: PLoS One. 2016 Oct 17;11(10):e0164792. doi: 10.1371/journal.pone.0164792 (PMC5066964; doi:10.1371/journal.pone.0164792)
Supplement: S3 File — (DOCX) [file pone.0164792.s007.docx]

**Supplemental File 2 (S2):** The details of 41 peer-reviewed journal articles and BCG-associated AEs identified from these articles

***Introduction***: In this supplemental file, we first provide a table that lists the PMIDs of these 41 peer-reviewed journal articles and the BCG-associated AEs identified from these articles. After this table is the detailed citation information of these 41 articles.

**The following table provides the article PMIDs and BCG-associated AEs identified from these articles:**

| **PMID** | **BCG usage** | **Adverse events** |
| --- | --- | --- |
| 26598141 | TB vaccination | musculoskeletal pain, erythema, induration, pruritus, injection site discharge, fever, headache, fatigue, gastrointestinal pain |
| 25890634 | TB vaccination | pruritus, hematoma, eczema, lymphadenitis, adenopathy, myalgia, paresthesia, rash, fever, headache, urticaria, asthenia, malaise, dizziness |
| 25475539 | TB vaccination | abscess, lymphadenopathy, fever |
| 25045811 | TB vaccination | lymphadenitis, fever, seizure, meningitis, kawasaki disease |
| 23230718 | TB vaccination | injection site ulcer, lymphadenitis, abscess |
| 21195982 | TB vaccination | axillary lymphadenitis |
| 26151543 | TB vaccination | lymphadenitis, osteitis, disseminated BCG infected |
| 26398442 | TB vaccination | vasculitis, purpura |
| 22300718 | TB vaccination | injection site ulcer, abscess |
| 25862299 | TB vaccination | erythema, injection site ulcer, induration, scar |
| 26259542 | TB vaccination | immune reconstitution inflammatory syndrome, disseminated BCG infection |
| 25409184 | TB vaccination | abscess, lymphadenitis, disseminated BCG infection, injection site ulcer, osteomyelitis |
| 22071248 | TB vaccination | lymphadenitis, injection site ulcer |
| 17140780 | TB vaccination | inflammation, discharge, injection site ulcer, lymphadenopathy |
| 18766374 | TB vaccination | injection site ulcer, abscess, lymphadenopathy, immune reconstitution inflammatory syndrome |
| 20799545 | TB vaccination | lymphadenitis |
| 12729348 | TB vaccination | injection site ulcer, lymphadenitis, acute cutaneous erythema |
| 11357213 | TB vaccination | abscess, injection site discharge, disseminated BCG infection, adenopathy, swelling, erythema, keloid |
| 19008268 | TB vaccination | keloid, axillary lymphadenitis, disseminated BCG infection |
| 16547086 | TB vaccination | abscess, suppurative adenitis, adenopathy |
| 25691177 | TB vaccination | induration, papule, pustule, injection site ulcer, scab, scar |
| 25887440 | TB vaccination | scar, C-reactive protein increased |
| 24679470 | TB vaccination | death |
| 25228607 | TB vaccination | lymphadenitis, hepatosplenomegaly, fever, lymphadenopathy |
| 21122568 | TB vaccination | disseminated BCG infection, death, eczema, hemorrhage, granuloma |
| 25173482 | TB vaccination | lymphadenitis, suppurative lymphadenitis |
| 25011356 | TB vaccination | fever, axillary lymphadenopathy, pneumonia, abscess, hepatosplenomegaly, osteomyelitis, hepatosplenic granuloma, hepatic abscess |
| 26632629 | bladder cancer treatment | granulomatous cystitis, macroscopic hematuria, fever |
| 26681820 | bladder cancer treatment | lower abdominal pain, cystitis, bladder atrophy, sepsis |
| 26605208 | bladder cancer treatment | cystitis, bacterial infection, macroscopic hematuria, sepsis, malaise, fever |
| 23452187 | bladder cancer treatment | cystitis, hematuria, malaise, fever, contracted bladder, granulomatous prostatitis, sepsis |
| 23181987 | bladder cancer treatment | urinary frequency, irritative bladder symptom, hematuria, fever, granulomatous, flu-like syndrome, malaise, sepsis, allergic reaction, hepatitis, pneumonitis |
| 25032208 | bladder cancer treatment | cystitis, pneumonitis, hepatitis, sepsis, pancytopenia |
| 22952293 | bladder cancer treatment | pollakiuria, macroscopic hematuria, dysuria |
| 22575238 | bladder cancer treatment | dysuria, bladder spasm, hematuria, bladder pain, incontinence, cystitis, nocturia, urethral pain, urinary tract infection, abdominal pain, asthenia, headache, kidney pain, malaise, diarrhea, rash |
| 26609439 | bladder cancer treatment | dysuria, fever, hematuria |
| 23829273 | bladder cancer treatment | hematuria, fever, chills, flu-like syndrome, lower urinary tract symptom |
| 23662001 | bladder cancer treatment | hematuria, pyuria, miction pain, fever, pollakiuria |
| 23910233 | bladder cancer treatment | bacterial cystitis, chemical cystitis, macroscopic hematuria, fever, lung infection, rash, malaise |
| 23858038 | bladder cancer treatment | arthralgia, fever, malaise, headache, hematuria |
| 22407891 | bladder cancer treatment | fever, malaise, C-reactive protein increased |

**The following is the detailed citation information of the 41 articles:**

***Note***: In references [[1-27](#_ENREF_1)], BCG was used for TB vaccination. In references [[28-41](#_ENREF_28)], BCG was used for bladder cancer treatment.

1. Spertini F, Audran R, Chakour R, Karoui O, Steiner-Monard V, et al. (2015) Safety of human immunisation with a live-attenuated Mycobacterium tuberculosis vaccine: a randomised, double-blind, controlled phase I trial. Lancet Respir Med 3: 953-962. ***PMID: 26598141***

2. Mayet A, Duron S, Meynard JB, Koeck JL, Deparis X, et al. (2015) Surveillance of adverse events following vaccination in the French armed forces, 2011-2012. Public Health 129: 763-768. ***PMID: 25890634***

3. Bonetto C, Trotta F, Felicetti P, Alarcon GS, Santuccio C, et al. (2015) Vasculitis as an adverse event following immunization - Systematic literature review. Vaccine. ***PMID: 26398442***

4. Blakney AK, Tchakoute CT, Hesseling AC, Kidzeru EB, Jones CE, et al. (2015) Delayed BCG vaccination results in minimal alterations in T cell immunogenicity of acellular pertussis and tetanus immunizations in HIV-exposed infants. Vaccine 33: 4782-4789. ***PMID: 26259542***

5. Dierig A, Tebruegge M, Krivec U, Heininger U, Ritz N, et al. (2015) Current status of Bacille Calmette Guerin (BCG) immunisation in Europe - A ptbnet survey and review of current guidelines. Vaccine 33: 4994-4999. ***PMID: 26151543***

6. Lee HS, Seo KJ, Kim JJ (2015) Chest wall granuloma associated with BCG vaccination presenting as hot abscess in an immunocompetent infant. J Cardiothorac Surg 10: 29. ***PMID: 25887440***

7. Geldenhuys HD, Mearns H, Foster J, Saxon E, Kagina B, et al. (2015) A randomized clinical trial in adults and newborns in South Africa to compare the safety and immunogenicity of bacille Calmette-Guerin (BCG) vaccine administration via a disposable-syringe jet injector to conventional technique with needle and syringe. Vaccine 33: 4719-4726. ***PMID: 25862299***

8. Faridi MM, Srivastava S (2015) Effect of simultaneous administration of oral polio vaccine on local reaction of BCG vaccine in term infants. Indian Pediatr 52: 115-118. ***PMID:*** ***25691177***

9. Clothier HJ, Hosking L, Crawford NW, Russell M, Easton ML, et al. (2015) Bacillus Calmette-Guerin (BCG) vaccine adverse events in Victoria, Australia: analysis of reports to an enhanced passive surveillance system. Drug Safety 38: 79-86. ***PMID:*** ***25475539***

10. Al-Hajoj S, Memish Z, Abuljadayel N, AlHakeem R, AlRabiah F, et al. (2014) Molecular confirmation of Bacillus Calmette Guerin vaccine related adverse events among Saudi Arabian children. PLoS One 9: e113472. ***PMID: 25409184***

11. Indumathi CK, Kowtal PM, Poornima RN, Lewin S (2014) Clinical profile and outcome of clinical BCG disease in infants. Indian Pediatr 51: 730-732. ***PMID:*** ***25228607***

12. Soh SB, Han PY, Tam KT, Yung CF, Liew WK, et al. (2014) Investigations into an outbreak of suppurative lymphadenitis with BCG vaccine SSI((R)) in Singapore. Vaccine 32: 5809-5815. ***PMID: 25173482***

13. Thoon KC, Soh SB, Liew WK, Gunachandran A, Tan NW, et al. (2014) Active surveillance of adverse events following childhood immunization in Singapore. Vaccine 32: 5000-5005. ***PMID: 25045811***

14. Mazzucchelli JT, Bonfim C, Castro GG, Condino-Neto AA, Costa NM, et al. (2014) Severe combined immunodeficiency in Brazil: management, prognosis, and BCG-associated complications. J Investig Allergol Clin Immunol 24: 184-191. ***PMID: 25011356***

15. Marciano BE, Huang CY, Joshi G, Rezaei N, Carvalho BC, et al. (2014) BCG vaccination in patients with severe combined immunodeficiency: complications, risks, and vaccination policies. J Allergy Clin Immunol 133: 1134-1141. ***PMID:*** ***24679470***

16. Krysztopa-Grzybowska K, Paradowska-Stankiewicz I, Lutynska A (2012) The rate of adverse events following BCG vaccination in Poland. Przegl Epidemiol 66: 465-469. ***PMID: 23230718***

17. Anderson EJ, Webb EL, Mawa PA, Kizza M, Lyadda N, et al. (2012) The influence of BCG vaccine strain on mycobacteria-specific and non-specific immune responses in a prospective cohort of infants in Uganda. Vaccine 30: 2083-2089. ***PMID:*** ***22300718***

18. Pereira SM, Barreto ML, Pilger D, Cruz AA, Sant'Anna C, et al. (2012) Effectiveness and cost-effectiveness of first BCG vaccination against tuberculosis in school-age children without previous tuberculin test (BCG-REVAC trial): a cluster-randomised trial. Lancet Infect Dis 12: 300-306. ***PMID: 22071248***

19. Santos A, Dias A, Cordeiro A, Cordinha C, Lemos S, et al. (2010) Severe axillary lymphadenitis after BCG vaccination: alert for primary immunodeficiencies. J Microbiol Immunol Infect 43: 530-537. ***PMID: 21195982***

20. Cheent K, Nolan J, Shariq S, Kiho L, Pal A, et al. (2010) Case Report: Fatal case of disseminated BCG infection in an infant born to a mother taking infliximab for Crohn's disease. J Crohns Colitis 4: 603-605. ***PMID: 21122568***

21. Al Awaidy S, Bawikar S, Prakash KR, Al Rawahi B, Mohammed AJ (2010) Surveillance of adverse events following immunization: 10 years' experience in Oman. East Mediterr Health J 16: 474-480. ***PMID:*** ***20799545***

22. Hawkridge A, Hatherill M, Little F, Goetz MA, Barker L, et al. (2008) Efficacy of percutaneous versus intradermal BCG in the prevention of tuberculosis in South African infants: randomised trial. BMJ 337: a2052. ***PMID: 19008268***

23. Fernandes RC, de Araujo LC, Medina-Acosta E (2009) Reduced rate of adverse reactions to the BCG vaccine in children exposed to the vertical transmission of HIV infection and in HIV-infected children from an endemic setting in Brazil. Eur J Pediatr 168: 691-696. ***PMID: 18766374***

24. Dommergues MA, de la Rocque F, Dufour V, Floret D, Gaudelus J, et al. (2007) [French survey about intradermal BCG SSI adverse events in children under 6 years of age]. Arch Pediatr 14: 102-108. ***PMID: 17140780***

25. Bolger T, O'Connell M, Menon A, Butler K (2006) Complications associated with the bacille Calmette-Guerin vaccination in Ireland. Arch Dis Child 91: 594-597. ***PMID: 16547086***

26. Dourado I, Rios MH, Pereira SM, Cunha SS, Ichihara MY, et al. (2003) Rates of adverse reactions to first and second doses of BCG vaccination: results of a large community trial in Brazilian schoolchildren. Int J Tuberc Lung Dis 7: 399-402. ***PMID:*** ***12729348***

27. Jeena PM, Chhagan MK, Topley J, Coovadia HM (2001) Safety of the intradermal Copenhagen 1331 BCG vaccine in neonates in Durban, South Africa. Bull World Health Organ 79: 337-343. ***PMID: 11357213***

28. Miyata Y, Sakai H (2015) Predictive Markers for the Recurrence of Nonmuscle Invasive Bladder Cancer Treated with Intravesical Therapy. Dis Markers 2015: 857416. ***PMID: 26681820***

29. Chou R, Buckley D, Fu R, Gore JL, Gustafson K, et al. (2015). Emerging Approaches to Diagnosis and Treatment of Non-Muscle-Invasive Bladder Cancer. Rockville (MD). ***PMID:*** ***26632629***

30. Kandeel W, Abdelal A, Elmohamady BN, Sebaey A, Elshaaer W, et al. (2015) A comparative study between full-dose and half-dose intravesical immune bacille Calmette-Guerin injection in the management of superficial bladder cancer. Arab J Urol 13: 233-237. ***PMID:*** ***26609439***

31. Decaestecker K, Oosterlinck W (2015) Managing the adverse events of intravesical bacillus Calmette-Guerin therapy. Res Rep Urol 7: 157-163. ***PMID: 26605208***

32. Marquez-Batalla S, Fraile-Villarejo E, Belhassen-Garcia M, Gutierrez-Zubiaurre N, Cordero-Sanchez M (2014) Disseminated infection due to Mycobacterium bovis after intravesical BCG instillation. World J Clin Cases 2: 301-303. ***PMID: 25032208***

33. Brausi M, Oddens J, Sylvester R, Bono A, van de Beek C, et al. (2014) Side effects of Bacillus Calmette-Guerin (BCG) in the treatment of intermediate- and high-risk Ta, T1 papillary carcinoma of the bladder: results of the EORTC genito-urinary cancers group randomised phase 3 study comparing one-third dose with full dose and 1 year with 3 years of maintenance BCG. Eur Urol 65: 69-76. ***PMID: 23910233***

34. Miyazaki J, Hinotsu S, Ishizuka N, Naito S, Ozono S, et al. (2013) Adverse reactions related to treatment compliance during BCG maintenance therapy for non-muscle-invasive bladder cancer. Jpn J Clin Oncol 43: 827-834. ***PMID:*** ***23858038***

35. Zhu S, Tang Y, Li K, Shang Z, Jiang N, et al. (2013) Optimal schedule of bacillus calmette-guerin for non-muscle-invasive bladder cancer: a meta-analysis of comparative studies. BMC Cancer 13: 332. ***PMID:*** ***23829273***

36. Inamoto T, Ubai T, Nishida T, Fujisue Y, Katsuoka Y, et al. (2013) Comparable effect with minimal morbidity of low-dose Tokyo 172 strain compared with regular dose Connaught strain as an intravesical bacillus Calmette-Guerin prophylaxis in nonmuscle invasive bladder cancer: Results of a randomized prospective comparison. Urol Ann 5: 7-12. ***PMID: 23662001***

37. Witjes JA, Palou J, Soloway M, Lamm D, Kamat AM, et al. (2013) Current clinical practice gaps in the treatment of intermediate- and high-risk non-muscle-invasive bladder cancer (NMIBC) with emphasis on the use of bacillus Calmette-Guerin (BCG): results of an international individual patient data survey (IPDS). BJU Int 112: 742-750. ***PMID: 23452187***

38. Kawai K, Miyazaki J, Joraku A, Nishiyama H, Akaza H (2013) Bacillus Calmette-Guerin (BCG) immunotherapy for bladder cancer: current understanding and perspectives on engineered BCG vaccine. Cancer Sci 104: 22-27. ***PMID: 23181987***

39. Kunieda F, Kitamura H, Niwakawa M, Kuroiwa K, Shinohara N, et al. (2012) Watchful waiting versus intravesical BCG therapy for high-grade pT1 bladder cancer with pT0 histology after second transurethral resection: Japan Clinical Oncology Group Study JCOG1019. Jpn J Clin Oncol 42: 1094-1098. ***PMID:*** ***22952293***

40. Dinney CP, Greenberg RE, Steinberg GD (2013) Intravesical valrubicin in patients with bladder carcinoma in situ and contraindication to or failure after bacillus Calmette-Guerin. Urol Oncol 31: 1635-1642. ***PMID:*** ***22575238***

41. Davies B, Ranu H, Jackson M (2012) Pulmonary complications of intravesicular BCG immunotherapy. Thorax 67: 933-934. ***PMID: 22407891***
